# Design and evaluation of a new mobile application to improve the management of minor ailments: a pilot study

**DOI:** 10.1186/s12913-022-08292-9

**Published:** 2022-07-15

**Authors:** Ehab Mudher Mikhael, Fadya Yaqoob Al-Hamadani, Ali Mohammed Hadi

**Affiliations:** 1 Clinical Pharmacy Department, College of Pharmacy, University of Baghdad, Baghdad, Iraq; 2 Clinical Pharmacy Department, College of Pharmacy, University of Basrah, Basrah, Iraq

**Keywords:** Mobile application, Minor ailments, Community pharmacists

## Abstract

**Background:**

Seeking pharmacist advice about minor ailments is a common practice among Iraqi patients because such advice is free and quick. Unfortunately, the assessment and management of minor ailments by Iraqi pharmacists were inappropriate. Therefore, this study aimed to develop a model for a mobile application that can assist community pharmacists in the diagnosis and management of minor ailments.

**Methods:**

The scientific content of the application was based on the information in the symptoms in the pharmacy and British National Formulary books. The design and content of the application were approved by two experts. Thereafter, the application was built for Android mobiles using flutter technology and dart language. A pre-post pilot study was conducted to assess outcomes associated with use of the application, including user acceptance and appropriateness of clinical recommendations. Fifteen students from the College of Pharmacy/University of Baghdad who had an Android mobile participated in this study. Two different scenarios about diarrhea were used during the pilot study, in which the researcher acted as a patient (SP) and the participant student as a pharmacist.

**Results:**

After using the application, the number of questions asked by the participated student to the SP was significantly increased to about double. Additionally, providing the SP with appropriate non-pharmacological and pharmacological therapy along with optimum counseling and education were also significantly improved. All study participants agreed on the application’s ease of use and ability to reduce diagnosis and medication errors.

**Conclusions:**

The implementation of the newly developed mobile application, diarrhea management step by step, was associated with improvements in assessment and recommended treatments for diarrhea cases with good acceptance by a pilot sample of pharmacy students at Baghdad University.

**Supplementary Information:**

The online version contains supplementary material available at 10.1186/s12913-022-08292-9.

## Introduction

In a community pharmacy, the pharmacist’s job has been shifted from just a medication dispenser to more sophisticated services. These services include reviewing the appropriateness of prescribed medications, providing healthcare services through counseling and educating patients about their treatment to enhance efficacy, safety and adherence to medications, and most commonly through the management of minor ailments [1]. According to the roles of the Iraqi Syndicate of pharmacists, pharmacists are allowed to manage minor ailments in the community setting by dispensing over-the-counter medications without the need for a physician’s prescription [2].

The pharmacist’s advice about minor ailments is free and quick; this is in contrast to the physician consultation, which requires the patient to pay money and wait for a long period [3]. Meanwhile, minor ailments services can reduce the high workload pressures on physician clinics, primary care clinics, and even hospitals [4] and thus reduce long-term healthcare costs [5]. Besides that, the abundance of community pharmacies in Iraq, especially in large towns such as Baghdad, Basrah, and Nineveh, can further encourage the Iraqi patient to request a pharmacist’s advice about minor ailments [6]. Meanwhile, asking for advice about minor ailments from community pharmacists is common among individuals in developing [7] and developed countries [8].

An initial response of the pharmacist in managing a minor ailment is by assessment of the patient’s case through gathering information [9]. Assessment of patients’ symptoms is highly appreciated because inappropriate and/or incomplete diagnosis of the case may render the pharmacist unable to detect alarming features and thus, consider the case to be a simple one and dispense some OTC medications to the patient; this action may ultimately harm the patient by delaying the correct diagnosis of the case until the patient experiences serious & sometimes irreversible complications [10, 11]. In this regard, several studies conducted in Iraq found that the majority of pharmacists fail to assess minor ailment cases properly using the WWHAM technique (W:who is the patient; W:what are the disease symptoms; H:how long is the disease; A:any action taken by the patient; and M: medication history) [12–14]. On the other hand, pharmacists must have good knowledge about medications to properly counsel and educate the patients about the dispensed treatment for minor ailments; however, this counseling role was shown to be poor among pharmacists in Iraq. In this regard, most pharmacists neglect the education of the patient about the dispensed medication, and if they do so, they focus on drug dosing frequency only [12, 15].

Some studies found that the use of technology such as computers [16] and smart mobiles [17] can assist pharmacists and improve their work efficiency and productivity [18] in issues of therapeutic drug monitoring and detecting drug-drug interactions, besides enhancing patients’ medication adherence [16, 17]. In Iraq, computers are mainly used to manage sales and financial issues in the pharmacy [6]. To our best knowledge, till now, no any mobile application was developed to assist pharmacists in decision making while managing minor ailments in community pharmacies [19]. Therefore, the current study aimed to develop a model for a new mobile application that can assist community pharmacists in the diagnosis and management of minor ailments by focusing on diarrhea as one of the commonest minor ailments [20].

## Methods

### Development of the assisting mobile application

**Fig. 1:**
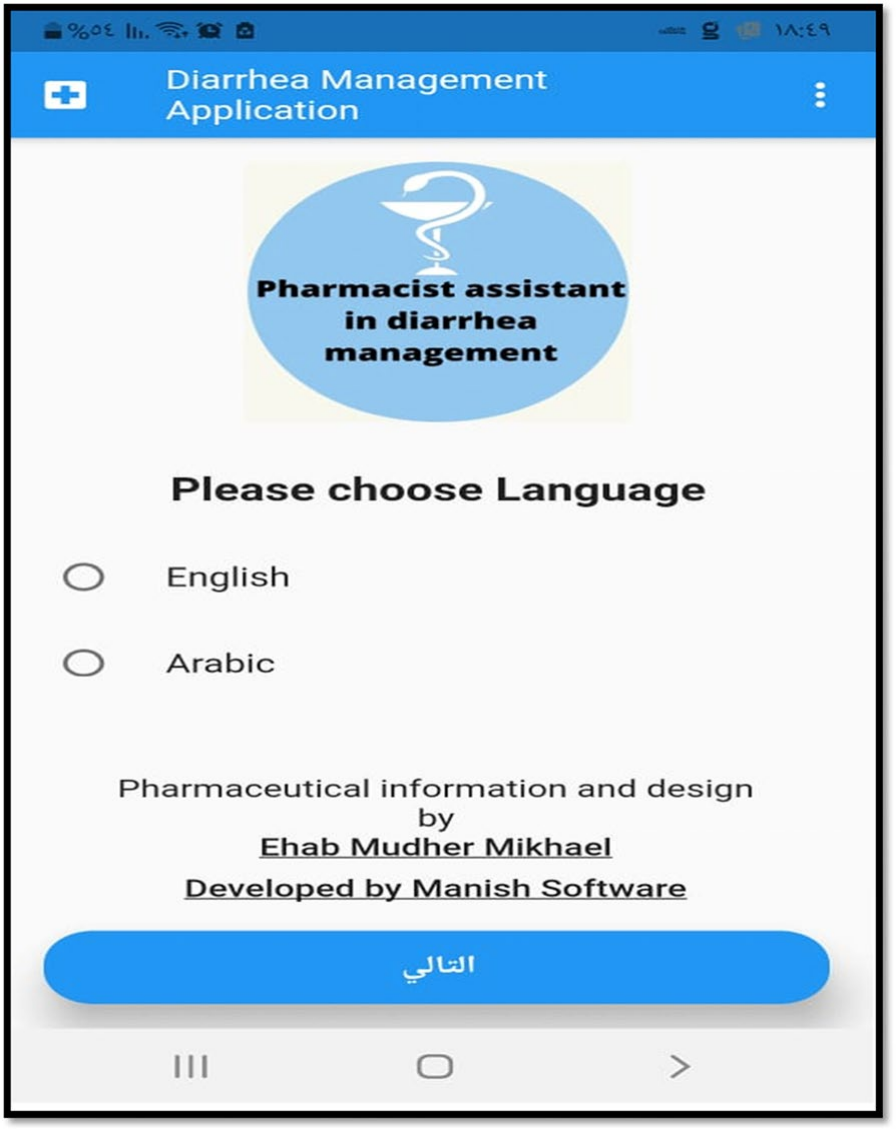
Screenshot of the first page of the application

The scientific content of the application was prepared by the main researcher according to the information in the latest version of the symptoms in the pharmacy [9] and British National Formulary [21] books. Then these contents were used to design the application in the following way (full details about the application design were given in appendix 1):

1. An introductory screen that includes a logo for the application and its aim “Pharmacist assistant in diarrhea management”, besides a simple list that allows the pharmacist to choose one of the available language options (Arabic and English) (Fig. 1).
2. Assessing the patient case through the use of WWHAM technique, besides some other specific questions like questioning about recent travel and antibiotic usage.
3. For minor cases that can be managed in the pharmacy:

A. Non-pharmacological treatment: The application was designed to present a specific non-pharmacological advice according to the patient’s age.
B. Pharmacological treatment: Oral rehydration solution (ORS) is the only treatment that the application presents for managing diarrhea among children younger than 12 years old. For patients older than 12 years, the application was designed to present a list of suitable medication(s) according to the patient’s age, symptoms, and medical and medication history. If more than one drug option appears on the screen, the application was designed to give a flexibility to the pharmacist to choose one drug option from the list according to the availability of products in his/her pharmacy and according to the patient preference regarding the medication cost and its dosing regimen.
C. Patient counseling and education: after choosing a treatment option by a pharmacist, the application was designed to present the most relevant and significant information about that medication (dose, dosing regimen, treatment time scale (TTS), side effects, and ancillary notes like preparation and storage recommendations) to assist the pharmacist in remembering the counseling and educational notes about the product that must be mentioned to the patient.

### Validation of the application contents and design

The application’s scientific contents and design were reviewed by two pharmacists independently. They had a Ph.D. degree in clinical pharmacy and more than 10 years of experience in providing community pharmacy services [22].

Both experts were asked to rate the comprehensiveness of each part of the application contents (The assessment, the non-pharmacological advice, the pharmacological treatment, and the patient education) on 4-point scales: strongly agree, agree, disagree, and strongly disagree. The experts were given the opportunity to write their comments about the need for additional information and on the language and jargon issues [22, 23]. On the other hand, experts were asked to rate the suitability of the application design using 4-point scale: strongly agree, agree, disagree, and strongly disagree. Any disagreement between experts was resolved through consensus-based discussion [24]. To calculate an overall Index of Content Validity (OICV), the total number of items ranked with agreement by both reviewers was divided by the total number of items. A CVI score of 80% is considered an acceptable value for validating the developed application [22, 24]. Both experts agreed (OICV = 100%) on the contents and design of the application.

### Building of the application

After the approval of the application contents and design, the application itself was constructed by a team of developers from Manish Software Company, India. All team members had at least 5 years of experience in developing mobile applications. The application was built for Android mobiles using flutter technology and the latest dart language (version 2.12 March, 2021). To check the progress in programming of the application, a weekly draft version of the application was built and sent by the developer team to the main researcher through Whatsapp to find out any scientific or technical errors. A Zoom meeting between the main researcher and the development team was conducted to discuss and solve all detected problems. This process was continued over 2 month period (from mid April to mid June 2021). To approve the full version of the application, the main researcher tested it by entering the data for more than 200 different cases of diarrhea, and the results were optimum for all cases. The approved version of the application was uploaded into the Google play-store on 12th July 2021, with the name (diarrhea management step by step).

The cost of developing this application was 3000$ (USD); however, the company offered a 70% discount for the researcher since he was a Ph.D. student.

### Pilot testing of the application

A pre-post pilot study was conducted to assess outcomes associated with the use of the application, including user acceptance and appropriateness of clinical recommendations. This pilot study was ethically approved by the ethical committee at the College of Pharmacy – University of Baghdad.

Students at the pharmacy college – University of Baghdad who had taken a community pharmacy coursework, such coursework is usually given to students during the 4th stage, were eligible to participate in this study. Thus, all students who passed from the 4th to the 5th stage with a score of at least 70 degrees in community pharmacy and had an Android mobile were invited to participate in this study (133 students); however, only 15 students (11 females and 4 males) signed the informed consent and thus included in this pilot study. The study was conducted from 30 August to 12 September 2021. Two different scenarios about diarrhea were used during the pilot study to assess the developed application, in which the researcher acted as a patient and the participant student as a pharmacist.

For assessment of the developed application, each participant was interviewed twice, one time before and the other after using the application. The interview process was conducted using the Google classroom. Details in both interviews were documented manually because of unavailability of a record option in the free meeting at Google classroom. A stopwatch was used to measure the time of the interview with each participant. The first interview was conducted from 30th August to 8th September 2021, and it involved the assessment of the participant’s role in the management of diarrhea based on his/her knowledge obtained from community pharmacy lectures and also from the mandatory summer pharmacy training. After finishing the first interview with all participants, the name of the application in the Google play store (Diarrhea management step by step) was shared in the classroom on 8th September 2021. Thereafter, the method of using the application and its unique features and abilities were explained by another Google classroom meeting on 9th September 2021. At the end of this meeting, an open discussion and training about the application were offered to solve any problems facing participants during their use of the application for the first time. Students were asked to train on the usage of the application before the last interview to be familiar with its usage. The last interview, conducted from 9th September to 12th September 2021, involved a second assessment of the participant’s role in the diagnosis and management of the diarrhea case while using the developed application. The data obtained during each interview was documented manually and then transferred to specially designed checklists (Table 1). Two academic clinical pharmacists with a good experience in community pharmacy issues were given the checklist separately and were asked to rate the contents of the checklist on 4-point scales: strongly agree, agree, disagree, and strongly disagree [22, 23]. Any disagreement between experts was resolved through consensus-based discussion [24]. Both experts agreed (OICV = 100%) on the contents and design of the checklist.

**Table 1:** Checklist for assessing pharmacists’ role in management of diarrhea

Parameter		Pre application	Post application
Diagnostic (assessment) questions	Who is the patient?		
	What are the disease symptoms?		
	How long is the duration of diarrhea?		
	What is the action taken by the patient?		
	What is the patient’s medical history?		
	What’s the patient’s medication history?		
	Recent history of antibiotic usage		
	Recent history of travel abroad		
Action of the pharmacist	Treatment of the case		
	Referral to the physician		
Advising the patient about the non pharmacological measures	Mentioned appropriately		
	Mentioned inappropriately		
	Not mentioned		
The dispensed product to treat diarrhea	Antimotility agent (appropriate treatment)		
	Products other than antimotility agents		
	Dispensing an additional product with the antimotility agent		
Appropriateness of the dispensed OTC medication (no contraindication and/or no drug interaction)	Appropriate		
	Inappropriate		
Patient education about drug dose	Mentioned appropriately		
	Mentioned inappropriately		
	Not mentioned		
Patient education about drug dosing frequency	Mentioned appropriately		
	Mentioned inappropriately		
	Not mentioned		
Patient education about the duration of taking the dispensed medication	Mentioned appropriately		
	Mentioned inappropriately		
	Not mentioned		
Patient education about the possible medication side effects	Mentioned appropriately		
	Mentioned inappropriately		
	Not mentioned		

Mentioned appropriately: the information was given to the SP was correct and complete; mentioned inappropriately: the information was given to the SP either incorrect and/or incomplete

Optimum assessment of the case was defined as asking all WWHAM questions besides all other specific questions about diarrhea. Appropriate treatment of diarrhea by the pharmacist was defined as the dispensing of an antimotility agent to the SP. On the other hand, appropriate antimotility agent is the one that is not contraindicated and/or not interacted with other medications used by the SP. Optimum care of the case was defined by optimum assessment and providing the SP with appropriate treatment (pharmacological and non pharmacological) along with counseling about the dispensed medical therapy.

At the end of the study (the last interview), each participant was asked to rate the application in regard to 5 different points (ease of use, reducing the time needed for management of diarrhea cases, reducing diagnostic errors, reducing medication errors, applicability in daily clinical practice) using a numerical scale from 1 to 10. Scores of 1 and 2 were considered as strong disagreement, scores of 3 and 4 as disagreement, scores of 5 and 6 as neutral, scores of 7 and 8 as agreement, and scores of 9 and 10 as strong agreement.

### Diarrhea scenarios

The scenarios were written by the main researcher and reviewed and approved by two pharmacists with PhD in clinical pharmacy and had a good experience in providing community pharmacy services. In the first scenario (appendix 2), two unique features of the application were tested: the first one was the ability of the application to work as a drug-drug interaction checker and thus excluding the anti-motility drug from the list of suitable medications if it interacted with the used medications by the patient (i.e., Co-phenotrope is interacted with amitriptyline); the second one was the ability of the application to check about pregnancy and breast feeding for women at child bearing age. Thus, a pharmacist was in need to ask the SP 11 questions to assess this case appropriately. On the other hand, Two other specific features of the application were tested in the second scenario (appendix 3); the first one was the ability of the application to check the possibility of drug-induced diarrhea (by linking the onset of developing diarrhea with the date of taking bisoprolol (concor®)), and the second one was the ability of the application to exclude a contraindicated drug from the list of suitable medications (Co-phenotrope is contraindicated in patients with prostate hypertrophy). Thus, a pharmacist was in need to ask the SP 8 questions to assess this case appropriately.

### Statistical analysis

Data input and analysis were done using the statistical package for the Social Sciences **(**SPSS) version 17. Categorical variables were presented as numbers and frequencies, while continuous variables were presented as mean ± standard deviation. McNemar’s Chi square test was used to test the significance of the difference among paired categorical variables, while Fisher exact test was used to test the significance in the difference among non-paired categorical variables. Shapiro Wilk test was used to test the normality of continuous variables. Paired T-test was used to determine the difference in mean for normally distributed continuous variables, while Wilcoxon Sign test was used for abnormally distributed data. *P* values less than 0.05 were considered significant.

## Results

### Assessment of the participants’ role in the diagnosis of diarrhea

Before using the application, the most commonly asked questions were: “Who is the patient? & how long is the duration of diarrhea?”. On the other hand, questioning the SP about action taken and the recent usage of antibiotics were the least asked questions. The average number of questions asked to the SP was 4.33 questions in both scenarios (4.33 out of 11 in the 1st scenario and 4.33 out of 8 in the 2nd scenario). However, no any participant asked all of the necessary questions to the SP. The diarrhea was not diagnosed in an optimum way in both scenarios. After the usage of the developed application, all WWHAM questions were improved. Besides that, the number of questions asked by the participated student to the SP was significantly increased to about double. Furthermore, the optimum assessment (diagnosis) of the diarrhea case was significantly increased from 0 to 86.7% in the first scenario and from 0 to 100% in the second scenario. Further details are shown in Table 2.

**Table 2:** Assessment of the participant’s role in the diagnosis of diarrhea

Diagnostic questions (Parameter)		Scenario 1			Scenario 2		
		Pre application N (%)	Post application N(%)	*P* value	Pre application N (%)	Post application N(%)	*P* value
Who is the patient?		10 (66.67)	14 (93.33)	0.134	12 (80)	15 (100)	0.248
What are the disease symptoms?		8 (53.33)	15 (100)	0.023	10 (66.67)	15 (100)	0.074
How long is the duration of diarrhea?		9 (60)	15 (100)	0.041	11 (73.33)	15 (100)	0.134
What is the action taken by the patient?		3 (20)	15 (100)	0.001	2 (13.33)	15 (100)	0.001
Questioning about medical history of the patient		2 (13.33)	15 (100)	0.001	10 (66.67)	15 (100)	0.074
Questioning about medication history of the patient		2 (13.33)	15 (100)	0.001	11 (73.33)	15 (100)	0.134
Additional conditions that necessitate referral	Recent history of antibiotic usage	2 (13.33)	15 (100)	0.001	3 (20)	15 (100)	0.001
	Recent history of travel abroad	5 (33.33)	15 (100)	0.004	7 (46.67)	15 (100)	0.013
Additional questions for scenario 1	Pregnancy	9 (60)	14 (93.33)	0.074			
	Breast feeding	5 (33.33)	14 (93.33)	0.004			
Number of questions asked by the pharmacist^a^		4.33 ± 2.32	10.73 ± 0.80	0.001	4.33 ± 1.40	8.00 ± 0.00	0.001
Optimum assessment of the case^b^		0 (0.0)	13 (86.67)	0.001	0 (0.0)	15 (100)	0.000

^a^Maximum number of necessary questions to the SP is 11 in the first scenario and 8 in the second scenario

^b^Optimum assessment of the case by asking all necessary questions to the SP, including WWHAM questions and diarrhea specific questions. Statistical analysis in this table was done through McNemar’s Chi Square test for paired categorical variables, and Wilcoxon signed rank test for continuous variables

### Assessment of the participant’s role in the treatment of diarrhea

Before using the developed application, all participated students dispensed a treatment to the diarrhea case, except two in the 2nd scenario, referred the SP to the physician. The non-pharmacological advice was mentioned appropriately to the SP by about 10% of the participated students. Antimotility agents were dispensed by 86.67% of the participated students in the 1st scenario and by 73.33% in the 2nd scenario. However, the choice of antimotility agent was appropriate only in 53.8% of cases for the 1st scenario and in 72.7% of cases for the second scenario. Appropriate treatment of diarrhea was dispensed by about half of the participants in both scenarios. After the usage of the application, mentioning an appropriate non-pharmacological advice and the choice of an appropriate antimotility were significantly improved in both scenarios. Additionally, no antibiotics were dispensed, and the overall number of dispensed products to treat the diarrhea case was reduced in both scenarios. Further details are shown in Table 3.

**Table 3:** Assessment of the pharmacist’s role in treatment of diarrhea

Parameter		Scenario 1			Scenario 2		
		Pre-application N(%)	Post-application N(%)	*P* value	Pre-application N(%)	Post-application N(%)	*P* value
Action of the pharmacist	Treatment of the case	15 (100)	15 (100)	1.0	13 (86.67)	15 (100)	0.480
	Referral to the physician	0 (0.0)	0 (0.0)		2^a^	0 (0.0)	
Advising the patient about the non pharmacological measures	Appropriate	2 (13.33)	14 (93.33)	0.002	1 (6.67)	15 (100)	0.001
	Inappropriate^b^	13 (86.67)	1 (6.67)		14 (93.33)	0 (0)	
The dispensed product to treat diarrhea^a^	Antimotility agent (appropriate treatment)	13 (86.67)	15 (100)	0.480	11 (73.33)	15 (100)	0.134
	Products other than antimotility (Antibiotics, laxatives, antiemetics)	2 (13.33)	0 (0.0)	0.480	2 (13.33)	0 (0.0)	0.480
	Dispensing an additional product (e.g ORS) with the antimotility agent	2 (13.33)	0 (0.0)	0.480	6 (40.0)	0 (0.0)	0.041
Appropriateness of the dispensed antimotility to the patient case (no contraindication or drug interaction)	Appropriate	7 (53.85)	15 (100)	0.000^	8 (72.73)	15 (100)	0.000^
	In appropriate	6 (46.15)	0 (0.0)		3 (27.27)	0 (0.0)	
Overall treatment of the case	No. of medications dispensed	1.13 ± 0.35	1.00 ± 0.00	0.157	1.46 ± 0.52	1.00 ± 0.00	0.014
	Appropriate treatment of the case^c^	7 (46.67)	14 (93.33)	0.023	8 (53.33)	15 (100)	0.023

^a^Some pharmacists dispensed more than one product for the simulated patient

^b^Inappropriate advice means either the advice was not mentioned or mentioned inappropriately (incomplete or wrong)

^c^Appropriate treatment of the case by dispensing antimotility agent without antibiotic. Statistical analysis in this table was done through McNemar’s Chi Square test for paired categorical variables, and Wilcoxon signed rank test for continuous variables

^calculations based on percentage values

### Assessment of the participant’s role in patient’s counseling and education about the prescribed antimotility medication

Before using the application, dose and dosing regimen were the most commonly mentioned information to the SP. In both scenarios, only 2 participants mentioned the treatment time scale (TTS), while no any participant mentioned drug side effects. All participated students failed to provide the SP with appropriate counseling and education. After the usage of the application, all points of patient education and counseling were improved; however, a significant improvement was detected only in regard to mentioning the TTS and drug side effects. Additionally, the ability of the participated student to provide the SP with appropriate counseling was significantly improved in both scenarios (66.67 and 86.67%, respectively). Further details are shown in Table 4.

**Table 4:** Assessment of the pharmacist’s role in patient’s counseling and education about the prescribed antimotility medication

Educational parameter		Scenario 1			Scenario 2		
		Pre-application (*N* = 13)	Post-application (*N* = 15)	*P* value	Pre-application (*N* = 11)	Post-application (*N* = 15)	*P* value
Initial dose	Mentioned appropriately N(%)	8 (61.54)	13 (86.67)	0.069	6 (54.55)	15 (100)	0.007
	Mentioned inappropriately N(%)	5 (38.46)	1 (6.67)		4 (36.36)	0 (0)	
	Not mentioned N(%)	0 (0)	1 (6.67)		1 (9.09)	0 (0)	
Dosing frequency	Mentioned appropriately N(%)	8 (61.54)	13 (86.67)	0.069	8 (72.73)	15 (100)	0.063
	Mentioned inappropriately N(%)	5 (38.46)	1 (6.67)		2 (18.18)	0 (0)	
	Not mentioned N(%)	0 (0)	1 (6.67)		1 (9.09)	0 (0)	
How long to take the medication (TTS)	Mentioned appropriately N(%)	2 (15.38)	12 (80)	0.001	2 (18.18)	15 (100)	0.000
	Mentioned inappropriately N(%)	5 (38.46)	0 (0)		2 (18.18)	0 (0)	
	Not mentioned N(%)	6 (46.15)	3 (20)		7 (63.64)	0 (0)	
Possible medication side effects	Mentioned appropriately N(%)	0 (0)	12 (80)	0.000	0 (0)	13 (86.67)	0.000
	Mentioned inappropriately N(%)	0 (0)	0 (0)		0 (0)	0 (0)	
	Not mentioned N(%)	13 (100)	3 (20)		13 (100)	2 (13.33)	
Overall patient education and counseling (dose, dosing regimen, side effect, TTS) (Max = 4 points)	All information that was given to the patient	2.62 ± 0.51	3.60 ± 0.63	0.006	2.25 ± 0.62	3.87 ± 0.35	0.004
	Information that was given to the patient in a correct way	1.46 ± 1.05	3.53 ± 1.06	0.002	1.42 ± 1.16	3.87 ± 0.35	0.005
Appropriate patient education and counseling N(%)		0 (0.0)	10 (66.67)	0.000	0 (0.0)	13 (86.67)	0.000

Mentioned appropriately: the information was given to the SP was correct and complete; Mentioned inappropriately: the information was given to the SP either incorrect and/or incomplete; Appropriate patient education and counseling means that the SP was provided with all four points of patient counseling and education in appropriate way. Statistical analysis was done through Fisher’s exact test for categorical variables, and Wilcoxon signed rank test for continuous variables

*TTS* Treatment time scale

### Assessment of the participant’s role in dealing with different diarrhea cases

Before using the application, the average time needed to deal with the diarrhea case was 102.6 seconds for the first scenario and 127.6 seconds for the second scenario. Besides that, all participants failed to provide an optimum care to diarrhea patient. After using the application, the average time needed to deal with the diarrhea case was increased in both scenarios; however, this increase was significant only in the first scenario. On the other hand, the pharmaceutical care provided to the SP with diarrhea (optimum assessment, choice of treatment, and patient counseling) was significantly improved after the use of the application in both scenarios (Table 5).

**Table 5:** Assessment of pharmacist’s role in management (dealing) with a diarrhea case

Parameter	Scenario 1			Scenario 2		
	Pre-application	Post-application	*P* value	Pre-application	Post-application	*P* value
Optimum care of the patient case a N(%)^a^	0 (0.0)	8 (53.33%)	0.023	0 (0.0)	13 (86.67)	0.001
Time to deal with the case Mean ± SD (Range) in seconds	102.6 ± 35.02 (57–163)	158.93 ± 41.89 (120–236)	0.001	127.6 ± 40.86 (43–181)	153.53 ± 31.70 (111–193)	0.057

^a^Optimum care of the case was defined by optimum assessment and providing the SP with an appropriate treatment (pharmacological and non pharmacological) along with counseling about the dispensed medical therapy. Statistical analysis was done through McNemar’s Chi Square test for categorical variables, and Paired T test for continuous variables

### Participants’ opinions about the developed application

More than 85% of participants strongly agreed with the application’s ease of use. About 47% of participants agreed strongly, and 40% of them only agreed with the ability of the application to reduce the time needed for dealing with diarrhea cases. All participants agreed on the importance of the application to reduce diagnosis and medication errors. In regard to the applicability of the developed application in daily pharmacy routine work, about 60% of participants felt that the application is applicable, whereas 40% were neutral in their opinion. Further details are shown in Table 6.

**Table 6:** Participants’ opinions about the developed application

Parameter	Strongly agree N(%)	Agree N(%)	Neutral N(%)	Disagree N(%)	Strongly disagree N(%)
Ease of use	13 (86.67)	2 (13.33)	0 (0.0)	0 (0.0)	0 (0.0)
Reduce time	7 (46.67)	6 (40)	2 (13.33)	0 (0.0)	0 (0.0)
Reduce diagnosis errors	15 (100)	0 (0.0)	0 (0.0)	0 (0.0)	0 (0.0)
Reduce medication errors	14 (93.33)	1 (6.67)	0 (0.0)	0 (0.0)	0 (0.0)
Applicability	1 (6.67)	8 (53.33)	6 (40)	0 (0.0)	0 (0.0)

## Discussion

The results of the current study showed that the most commonly asked question by pharmacy students was the duration of diarrhea, whereas questioning about action taken and a recent history of antibiotic usage were the least asked questions. A similar result was obtained by the final year MPharm students during the OSCE examination on diarrhea cases [25]. Meanwhile, such results were expected since human beings can forget the studied information rapidly [26]. In spite of the shortcoming of questioning skills, most students were able to reach a correct diagnosis of the case, and only two students referred the SP to the physician in the second scenario. This outcome was close to that obtained during the OSCE examination for MPharm students on diarrhea [25].

On the other hand, the number of questions asked by the participated student to the SP, and thus optimum assessment of the diarrhea case, was increased significantly to double after the usage of the developed application. Similarly, a computerized pharmacy decision support system was effective in doubling the number of questions asked by the pharmacist to patients with minor ailments (allergic rhinitis and conjunctivitis) [27]. Furthermore, the ability of the application to enhance the assessment of the case was mentioned by all participated students who strongly agreed with the application’s ability to reduce diagnosing errors.

The results of this study showed that 86.7% of participants treated the simulated case (scenario 2) without referral before using the application. On the other hand, all participants did not refer the SP (scenario 2) to the physician after using the application. Despite this benefit by the application, such a benefit was not significant. The possible explanation for lacking significance may be due to the fact that the used scenarios in the current study were designed to assess the pharmacist’s role in the management of diarrhea by presenting a simple case of diarrhea that can be managed in a pharmacy without the need for referral.

The results of the current study showed that about half of the participated students neglected the provision of the SP with non-pharmacological advice. On the other hand, the majority of participants who advised the SP with non-pharmacological measures did so either incompletely or incorrectly. This problem may be attributed to the lack of sufficient knowledge about the needed non-pharmacological measures to manage diarrhea and other minor ailments. Similarly, few of the Indonesian pharmacy students mentioned non-pharmacological advice for patients with cough [28]. Meanwhile, the advice about lifestyle measures was mentioned by nearly all the participated students after they used the application. This significant change could be attributed to the application’s ability in enhancing the knowledge of its users [29] or at least prevent the forgotten of information while dealing with the SP.

Regarding the treatment of diarrhea, the current study showed that appropriate treatment of diarrhea was recommended by about half of the participated students before they started the usage of the developed application. This was mainly attributed to the poor assessment of the SP case [30, 31]. On the other hand, a correct treatment was dispensed by nearly all participants after the usage of the developed application; this benefit may be linked to the ability of the application to enhance the assessment process of the patient case [32]. Thus, most of the participants expressed a strong agreement about the ability of the application to reduce medication errors.

Regarding patient education before using the application, the current study showed that all participants failed to provide the SP with appropriate counseling and education about the dispensed product in both scenarios. This poor patient education may result in inappropriate product usage and thus may harm the patient [10]. It is unclear whether this problem was due to the lack of knowledge about the recommended product details or to the forgetfulness of such details. On the other hand, the usage of the application resulted in significant improvement in patient education and counseling. This improvement was expected since the application was supplied with the scientific information about all anti-diarrheal medications, such as drug dose, dosing regimen, duration of treatment, and possible side effects.

The present study showed that the significant improvement in diarrhea management by the developed application was at the expense of increasing the time needed to deal with such cases. This increase in time was unexpected since the application was designed to prevent wasting pharmacists’ time by rapidly reminding them with the necessary assessment questions and drug information. Meanwhile, the significant increase in the number of questions asked and the given drug information by the participated students to the SP after the usage of the application may be the main reason for such an increase in time. This explanation is further confirmed by the fact that the increase in time was significant in the first scenario and was not significant in the second scenario. This may mean that the time increase may not always be the case while using the application (especially in cases that require referral). That’s why more than 86% of the participated students agreed to the ability of the application to reduce the time needed to deal with diarrhea cases. Besides that, 60% of the participants agreed on the applicability of the application for use in the daily routine work of a community pharmacy.

There are many limitations in the current study. The first one is the small sample size. The second one is the channeling bias, as only 15 of 133 eligible students were accepted to participate in this study. The third one is the testing of the application on senior pharmacy students and not on pharmacists. The fourth one is the possibility of performance bias because the researcher was the standardized patient in both the pre-app and post-app scenarios. The final limitation is the possibility of bias in assessing students’ responses because the researcher acted as the SP and as the investigator at the same time.

Therefore, it is highly recommended to perform a randomized controlled trial on a large sample of pharmacists to confirm the results of this pilot study and determine the benefit of the developed application in clinical practice.

## Conclusion

The implementation of the newly developed mobile application, diarrhea management step by step, was associated with improvements in assessment and recommended treatments for diarrhea cases with good acceptance by a pilot sample of pharmacy students at Baghdad University.

## Supplementary Information

**Additional file 1.**

## Data Availability

The datasets used and/or analysed during the current study available from the corresponding author on reasonable request.
