# Supplementary material for: Design and evaluation of a new mobile application to improve the management of minor ailments: a pilot study
Source: BMC Health Serv Res. 2022 Jul 15;22:920. doi: 10.1186/s12913-022-08292-9 (PMC9287937; doi:10.1186/s12913-022-08292-9)
Supplement: Supplementary file 1 — Additional file 1. [file 12913_2022_8292_MOESM1_ESM.docx]

Appendix 1: The design of the diarrhea management step by step application

1. An introductory screen that includes a logo for the application and its aim "Pharmacist assistant in diarrhea management", besides a simple list that allows the pharmacist to choose one of the available language options (Arabic and English).
2. Assessing the patient case through the use of WWHAM technique (Who is the patient? What are the symptoms? How long? Action taken; and Medication taken (and medical history), besides some other specific questions like questioning about recent travel and antibiotic usage (7).
3. Who is the patient? This part included questions about the patient gender and age. If the patient is a woman with child bearing age, then the application reminds the pharmacist to ask about pregnancy and breastfeeding status.
4. Do the patient using antibiotics at the current time or at least in the last week? This question was designed to appear immediately after the question of who is the patient, to allow the pharmacist to refer all antibiotic-induced diarrhea cases without the need to ask further questions, and thus saving the pharmacist time.
5. Is the patient recently returned from a travel? This question was also placed before the remaining WWHAM questions to allow the pharmacist to detect and refer travelers' diarrhea cases without the need to ask further questions, and thus saving the pharmacist time.
6. How long is the duration of the patient symptoms? The application arranged in a way to allow the pharmacist to choose the duration of the diarrhea from one of 4 possible answers (less than 1 day; 1 day- less than 2 days; 2 days – less than 3 days; 3 days or more). Besides that, the application was designed to make a quick decision for either referral of the case or continue with the remaining WWHAM questions treatment of the case after linking the duration of the diarrhea with age of the patient.
7. What are the symptoms? This part included a list of all possible symptoms of diarrhea. The application was arranged in a way that allows the pharmacist to choose one or more of the diarrhea-associated symptoms according to what symptoms are mentioned (suffered) by the patient. Besides that the application was developed to detect any alarming features of diarrhea (e.g., severe abdominal pain, severe vomiting, and/or blood in stool) that merit physician consultation; if such symptoms detected, the application shows a referral message and remind the pharmacist with the reason for referral to be able to explain that for the patient.
8. Medication taken: This part of the application was designed to include question about the medical and medication history of the patient. The application was supplied with a checklist for all possible diseases that may provoke diarrhea or their treatment can either induce diarrhea or interact with antimotility agents. The application designed to present a referral message if one of the following diseases were chosen:
9. Bowel tumor
10. Ulcerative colitis
11. Crohn's disease
12. Diverticulosis
13. Celiac disease

Regarding medication history, the application was fed with data about all medications that can induce diarrhea. If one or more of these medications was chosen, the application reminds the pharmacist to ask about the duration of using that medication(s). In case of using the medication recently, the application was designed to present a referral message due to the possibility of drug-induced diarrhea; otherwise drug-induced diarrhea is unlikely and thus the process of patient assessment continued.

1. Action taken: This part of the application was arranged in a way to let the pharmacist choose one of the possible actions for managing diarrhea (i.e., no action, dietary modifications, using specific herbs, taking some medications). The application was designed to go directly to the diarrhea treatment section, if no action is done or in case of consuming herbs or specific foods to manage the diarrhea, because there was no evidence for their benefits. Only if a patient is taking a medication, the application was designed to include some other questions to assess the suitability of the chosen medication and its dose in diarrhea management according to the patient case. That's why questioning about medication and medical history was placed before the questioning about action taken to allow the application to assess the suitability of the taken medication (i.e., free from contraindication and drug interaction) by the patient to manage his/her diarrhea. If an error is detected, the application can assist the pharmacist in his/her role by presenting a message that explain whether the problem in the appropriateness of the medication (drug interaction or contraindication) or in its dose, and also mention a recommended dose and duration of treatment. But if the chosen medication and its dose were suitable to manage diarrhea, then the application designed to present a question about the duration of using the treatment on the mobile screen; the answer for this question has 2 possibilities. If the period of taking the medication is longer than the treatment time scale of the diarrhea (1 day for children and 2 days for adults), then the application was designed to present a referral message because of the possibility of failed treatment. If the period of taking the medication is less than the treatment time scale of the case (1 day for children and 2 days for adults), then the application was designed to present a message that explain the necessity to continue the treatment and if no improvement within specific period of time (treatment time scale), then referral is needed.
2. For minor cases that can be managed in the pharmacy:
3. Non-pharmacological treatment: The application was designed to present a specific non-pharmacological advice according to the patient age. For infants the advice is limited to the need to continue feeding (whether breast feeding or bottle feeding) to prevent dehydration and malnutrition since milk is the major source of energy, fluid, and nutrients for infants. For patients > 1 year, the application was designed to present two advices; one about the necessity to drink plenty of clear, no milky fluids, and the other about the need to continue eating usual diet while avoiding fatty and sweet foods (foods with add sugar) as they can aggravate diarrhea by osmotic shifts.
4. Pharmacological treatment: Oral rehydration solution (ORS) is the only treatment that the application presents for managing diarrhea among children younger than 12 years old. Meanwhile the application was designed to give flexibility to the pharmacist to choose the ORS preparation (powder, or premixed solution) according to the availability of such products in his/her pharmacy and according to customer preference regarding the product's cost and its flavor. For patients older than 12 years, the application was designed to present a list of suitable medication(s) according to the patient's age, symptoms, and medical and medication history. In cases at which more than one drug option appears on the screen, the application was designed to give a flexibility to the pharmacist to choose one drug option from the list according to the availability of products in his/her pharmacy and according to the patient preference regarding the medication cost and its dosing regimen.
5. Patient counseling and education: after choosing a treatment option by a pharmacist, the application was designed to present the most relevant and significant information about that medication (dose, dosing regimen, treatment time scale, side effects, and ancillary notes like preparation and storage recommendations) to assist the pharmacist in remembering the counseling and educational notes about the product that must be mentioned to the patient.

Appendix 2: The first scenario

| Speaker | Requests and answers |
| --- | --- |
| SP | I want a strongest treatment for diarrhea |
| Pharmacist | For whom you want this treatment? |
| SP | To my wife |
| Pharmacist | How old is she? |
| SP | She is 30 years old |
| Pharmacist | Is she pregnant? |
| SP | No |
| Pharmacist | Is she breastfeeding? |
| SP | No |
| Pharmacist | How long does she suffer from diarrhea? |
| SP | About 1 day |
| Pharmacist | What are the symptoms that the patient suffers from along with diarrhea? |
| SP | She has some nausea and mild abdominal pain |
| Pharmacist | Does she suffer from other diseases? |
| SP | Yes, she has a Rheumatological disease (disc prolapse) |
| Pharmacist | What does the patient take for her disc prolapse? |
| SP | She takes amitriptyline according to the physician advice |
| Pharmacist | Did the patient take any action to manage diarrhea? |
| SP | No, she didn't |
| Pharmacist | Does the patient return from a travel recently? |
| SP | No |
| Pharmacist | Is the patient currently using any antibiotic or used antibiotic in the last week? |
| SP | No |

SP=Simulated patient

Appendix 3: The second scenario

| Speaker | Requests and answers |
| --- | --- |
| SP | I want the strongest treatment for diarrhea |
| Pharmacist | For whom you want this treatment? |
| SP | To my father |
| Pharmacist | How old is he? |
| SP | He is 60 years old |
| Pharmacist | How long does he suffer from diarrhea? |
| SP | Since yesterday night (about 1 day) |
| Pharmacist | What are the symptoms that the patient suffers from along with diarrhea? |
| SP | He has some abdominal pain |
| Pharmacist | Does she suffer from other diseases? |
| SP | Yes, he has hypertension and benign prostatic hyperplasia |
| Pharmacist | What does the patient take for his diseases? |
| SP | He takes Concor ® 2.5mg once daily for hypertension and Prostacare ® for prostatic hyperplasia |
| Pharmacist | When did the patient start taking Concor? |
| SP | The patient started using Concor about 2 years ago. |
| Pharmacist | Did the patient take any action to manage diarrhea? |
| SP | Yes, he eats a bitter tea |
| Pharmacist | Does the patient return from a travel recently? |
| SP | No |
| Pharmacist | Is the patient currently using any antibiotic or used antibiotic in the last week? |
| SP | No |

SP= Simulated patient
